# Cell-Cycle Analyses Using Thymidine Analogues in Fission Yeast

**DOI:** 10.1371/journal.pone.0088629

**Published:** 2014-02-13

**Authors:** Silje Anda, Erik Boye, Beata Grallert

**Affiliations:** Department of Cell Biology, Institute for Cancer Research, Oslo University Hospital, Oslo, Montebello, Norway; St. Georges University of London, United Kingdom

## Abstract

Thymidine analogues are powerful tools when studying DNA synthesis including DNA replication, repair and recombination. However, these analogues have been reported to have severe effects on cell-cycle progression and growth, the very processes being investigated in most of these studies. Here, we have analyzed the effects of 5-ethynyl-2′-deoxyuridine (EdU) and 5-Chloro-2′-deoxyuridine (CldU) using fission yeast cells and optimized the labelling procedure. We find that both analogues affect the cell cycle, but that the effects can be mitigated by using the appropriate analogue, short pulses of labelling and low concentrations. In addition, we report sequential labelling of two consecutive S phases using EdU and 5-bromo-2′-deoxyuridine (BrdU). Furthermore, we show that detection of replicative DNA synthesis is much more sensitive than DNA-measurements by flow cytometry.

## Introduction

Understanding the mechanisms of cell-cycle regulation and the maintenance of genomic integrity is a major objective of cancer research. Recent studies have revealed that cancer cells frequently suffer from enhanced replication stress, a fact that highlights the importance of understanding the mechanisms regulating DNA replication and DNA repair. A powerful tool for monitoring and quantifying DNA replication, repair and recombination is to label the DNA with nucleoside analogues [1]–[7]. Examples of such analogues are 5-bromo-2′-deoxyuridine (BrdU), 5-Chloro-2′-deoxyuridine (CldU), 5-Iodo-2′-deoxyuridine (IdU), and 5-ethynyl-2′-deoxyuridine (EdU). However, the presence of these thymidine analogues can lead to mutations, DNA damage and cell-cycle delay [8]. These complications occur for at least two reasons: (i) changing the dNTP pools is mutagenic and can lead to cell-cycle arrest [9]–[13] and (ii) thymidine analogues are mutagenic when incorporated into the DNA [14]. *In vivo* labelling of the DNA using thymidine analogues may perturb the very process under study and cell-cycle analyses depend critically on a minimum disturbance of the cell cycle itself. Therefore, choosing the appropriate analogue and protocol for an experiment requires careful consideration of the effects that the analogue may have on cell-cycle progression, how it might affect the experiment and the sensitivity of detection. In this work we have studied these parameters in the fission yeast *Schizosaccharomyces pombe*.

*S. pombe* is an excellent model organism for studies of DNA replication and the cell cycle. Labelling of the DNA with thymidine analogues has been used successfully in this organism, although not extensively. The limited application may stem from the fact that fission yeast does not naturally take up exogenous nucleosides from the surrounding medium, nor does it contain the salvage pathway of nucleotide synthesis that would allow phosphorylation of deoxyribonucleosides. Expressing the human Equilibrative Nucleoside Transporter (hENT1) and the Herpes Simplex virus thymidine kinase (*hsv-tk*) in fission yeast allows both uptake and efficient intracellular phosphorylation of thymidine analogues [4], [7]. There are two independent strains available carrying the hENT1 transporter and thymidine kinase (Table 1); one constructed by the Rhind lab [7] and another one constructed by the Forsburg lab [4]. Using these strains, the DNA has been successfully labelled with BrdU, CldU, IdU and EdU [3]–[5], [7], [15]. However, there are studies suggesting that BrdU and EdU incorporation affects cell-cycle progression and viability also in fission yeast cells [7], [15], [16]. It was recently shown that labelling the DNA of fission yeast with BrdU activates the DNA damage checkpoint [16], like it does in mammalian cells. In this study we have improved and refined the use of thymidine analogues to allow their detectable labelling in fission yeast cells with a minimum of cell-cycle perturbation. We have addressed which analogue is best for cell-cycle analyses, how sensitive the method is and how to double-label the DNA with two different analogues.

**Table 1:** Strains used in this study.

Strain number	Genotype	Derives from	Reference
1402	leu1-32::hENT1-leu1+ (pJAH29) ura4-294::hsv-tk-ura4+(pJK210-tk+) ade6-704 h-	Forsburg strain	Hodson et al. 2003 [4]
1848	cdc10-M17 leu1-32::hENT1-leu1+ (pJAH29) ura4-294::hsv-tk-ura4+ (pJK210-tk+) ade6-704	Forsburg strain	This study, derives from 1402
1495	cdc10-M17 sep1:HBD:kanMX6 leu1-32::hENT1-leu1+(pJAH29) ura4-294::hsv-tk-ura4+ (pJK210-tk+) h-	Forsburg strain	This study, derives from 1402
1947	leu1-32 ura4-D18 ade6-210 his7-366 leu1::pFS181(leu1adh1:hENT1) pJL218 (his7 adh1:tk) h-	Rhind strain	Sivakumar et al. 2004 [7]
1961	cdc10-M17 leu1-32 ura4-D18 his7-366 leu1::pFS181(leu1adh1:hENT1) pJL218 (his7 adh1:tk)	Rhind strain	This study, derives from 1947

## Materials and Methods

### Yeast Strains and Growth Conditions

All strains used carry a *cdc10-M17* mutation and the hsv-tk and hENT1 genes (see Table 1). Strain construction and maintenance were as described [17]. The cells were grown in Yeast Extract medium (YES) or Edinburgh Minimal Medium (EMM) at 25°C. The cells were synchronized in G1 phase by incubating the *cdc10-M17* mutants at 36°C for 3 hours (YES) or 4 hours (EMM) before releasing them into the cell cycle at 25°C.

### EdU Incorporation and Detection

Cells grown in YES were synchronized in G1 phase and released in the presence of 10 µM EdU. The cells were fixed in 70% ethanol at the time points indicated, washed once with PBS containing 2% Fetal Calf Serum (FCS) (Gibco), 0.05% Tween-20 (Sigma-Aldrich), and treated with 1 mg/ml zymolyase 20T (Sunrise Science Products) for 20 minutes at 36°C. The cells were washed once with PBS and permeabilized with 1% triton for 1 minute. For EdU detection, the Click-IT EdU Alexa Flour 488/555 kit (Life Science) was used as described by the manufacturer. For analyses by immunoflourescence microscopy, cells were mounted on poly-L-lysine microscope slides, dried, and viewed in the presence of 0.2 µg/ml 4′,6-diamidino-2-phenylindole (DAPI). Images were collected by a Leica CTR DM6000 microscope with a Leica DFC350FX camera.

### CldU Incorporation and Detection

Cells grown in YES were synchronized in G1 phase and released in the presence of 95 µM CldU. After adding the analogue, the cells were incubated in the dark until they were fixed. Cell fixation and zymolyase treatment were as described above, the cells were treated with 4M HCl for 10 minutes, washed three times with PBS, and incubated for 1 hour in PBS, 10% FCS and 0.05% Tween-20. Primary antibody against CldU (BU/175, Abcam, cat.# 7384) was added at a dilution of 1∶2000, and the cells were incubated overnight at 4°C on a rotating wheel. The next day, the cells were washed 3 times with PBS, 2% FCS and 0.05% Tween-20. Secondary anti-rat IgG:Alexa Fluor 568 (Invitrogen cat. #A11077) was added at a dilution of 1∶250. After incubation for 2 hours at room temperature, the cells were washed 3 times with PBS, 2% FCS and 0.05% Tween-20. The cells were mounted and viewed as above.

### EdU/BrdU Incorporation and Detection

Cells grown in YES were synchronized in G1 phase and released in the presence of 10 µM EdU. After the first S phase, EdU was removed by washing the cells three times with equal volumes of YES. Before the second S phase 50 µM BrdU was added and kept in the medium until the second S phase was completed. After adding the analogue the cells were incubated in the dark until they were fixed. Cell fixation, zymolase- and HCl-treatment and blocking were as described above. EdU detection was then performed as described above. Primary antibody against BrdU (Invitrogen cat # B-35130, MoBU1) was used at a dilution of 1∶20 and the cells were incubated overnight at 4°C on a rotating wheel. The next day, the cells were washed 3 times with PBS, 2% FCS and 0.05% Tween-20. Secondary anti-mouse IgG1:FITC (AbD Serotec cat.# STAR132F) was added at a dilution of 1∶250. After incubation for 2 hours at room temperature, the cells were washed 3 times with PBS, 2% FCS and 0.05% Tween-20. The cells were mounted and viewed as above.

### Mitotic Index

Cells were fixed in 70% ethanol, washed 3 times with PBS and stained with DAPI before being visualized using the Leica DM6000 microscope. Cells were scored as mitotic when they were binucleates with no septum.

### Binucleate Index

Cells were fixed with 70% ethanol and processed for Sytox Green staining. Binucleate cells were quantified by flowcytometry as described [18].

### Hydroxyurea Block-and-release

Cells grown in YES were synchronized in G1 phase and released in the presence of 10 µM EdU with or without 15 mM hydroxyurea (HU). Samples were harvested at shift-down to 25°C and after 50 minutes. Sample treatment and EdU detection was performed as described above.

### UV Irradiation

Cells growing in EMM were UV-irradiated (254 nm) in a thin layer of EMM, under continuous stirring, with a dose of 1100 J/m^2^ (10–20% survival in G1; >90% survival in asynchronous cells) as described [19].

### CPD Detection

Cells growing in EMM were UV-irradiated as described above and samples were harvested at the indicated time points. Cell were fixed in 70% ethanol at −20°C and sample processing was performed the same way as described for the CldU detection. Cells were incubated overnight with an anti-CPD antibody (abcam ab10347), in a 1∶750 dilution. The next day the cells were washed 3 times using PBS and incubated for 2 hours with a CY3-conjugated secondary anti-mouse antibody (1∶250). The cells were then washed three times, mounted and visualised as described.

### Flowcytomery

Cells grown in YES were synchronized in G1 phase, released and harvested every 10 minutes. The samples were prepared as described [19] and DNA content was measured using a Becton-Dickinson LSRII flow cytometer. The results were analysed and quantified as previously described [18].

### Survival Assay

Cells growing in EMM were synchronized in G1 phase and released in the presence of 10 µM EdU or 50 µM CldU. The analogues were removed from the medium after 1 or 3 hours by washing 3 times with equal volumes of medium. The cells were then plated onto YES plates in 2 × serial dilutions and the plates were incubated at 25°C for 3 days. The cells labelled for 1 hour were incubated for a total of 4 hours before plated.

## Results and Discussion

### Optimizing the Labelling

High levels of thymidine analogues are known to arrest or delay the cell cycle, leading to elongated cells, presumably due to checkpoint activation. The cell-cycle effects after labelling the DNA with thymidine analogues might depend on both the duration of labelling and the concentration of the analogue [4], [7]. Here we have optimized both of these parameters for cell-cycle analyses. We used the strain deriving from the Forsburg lab for most of these analyses and also compared the strains constructed by the Forsburg and Rhind labs in some of the experiments. The strains used in this study, as well as their origin, are listed in Table 1.

**Figure 1:**
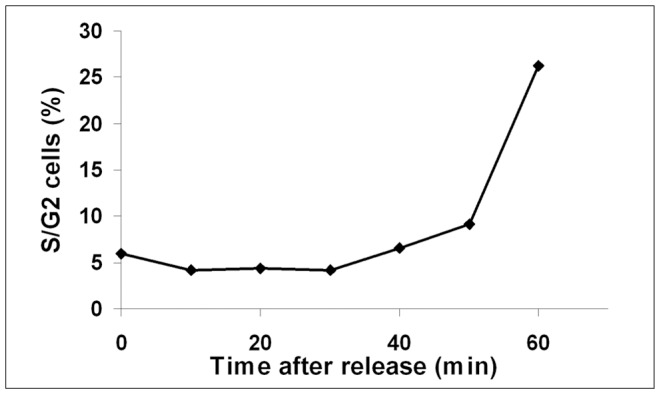
Timing of S phase using flow cytometry. Cell-cycle kinetics of cells arrested in G1 phase and released into the cell cycle. Samples were harvested, fixed and processed as described, and analysed by flow cytometry (10 000 cells. The graph shows the percentage of cells in S and G2 phase at the indicated time points.

If the cells are affected by prolonged exposure to the analogues it would be advantageous to minimize the time the analogues are present in the medium and to ensure that the label is only present during DNA replication. We set out to determine the optimal CldU-concentration that allows detection of DNA labelling but avoids severe effects on the cell-cycle progression. G1-synchronized cells were released into YES medium containing 760, 380, 190, 95, 50, 10 µM or no CldU. Without any analogue present, the start of S phase could be detected by flow cytometry 40–50 minutes after release, as an increase in cellular DNA content (Fig. 1, Fig. S1). All the concentrations used, with the exception of 10 µM, proved to be sufficient to detect DNA synthesis by fluorescence microscopy, since the cells showed about the same signal intensity for all concentrations from 50 µM and higher (Fig. S2). We conclude that 50 µM CldU administered for 1 hour after G1-synchronization is sufficient to detect replicative DNA synthesis.

### Short-term Effects of EdU- and CldU-labelling

**Figure 2:**
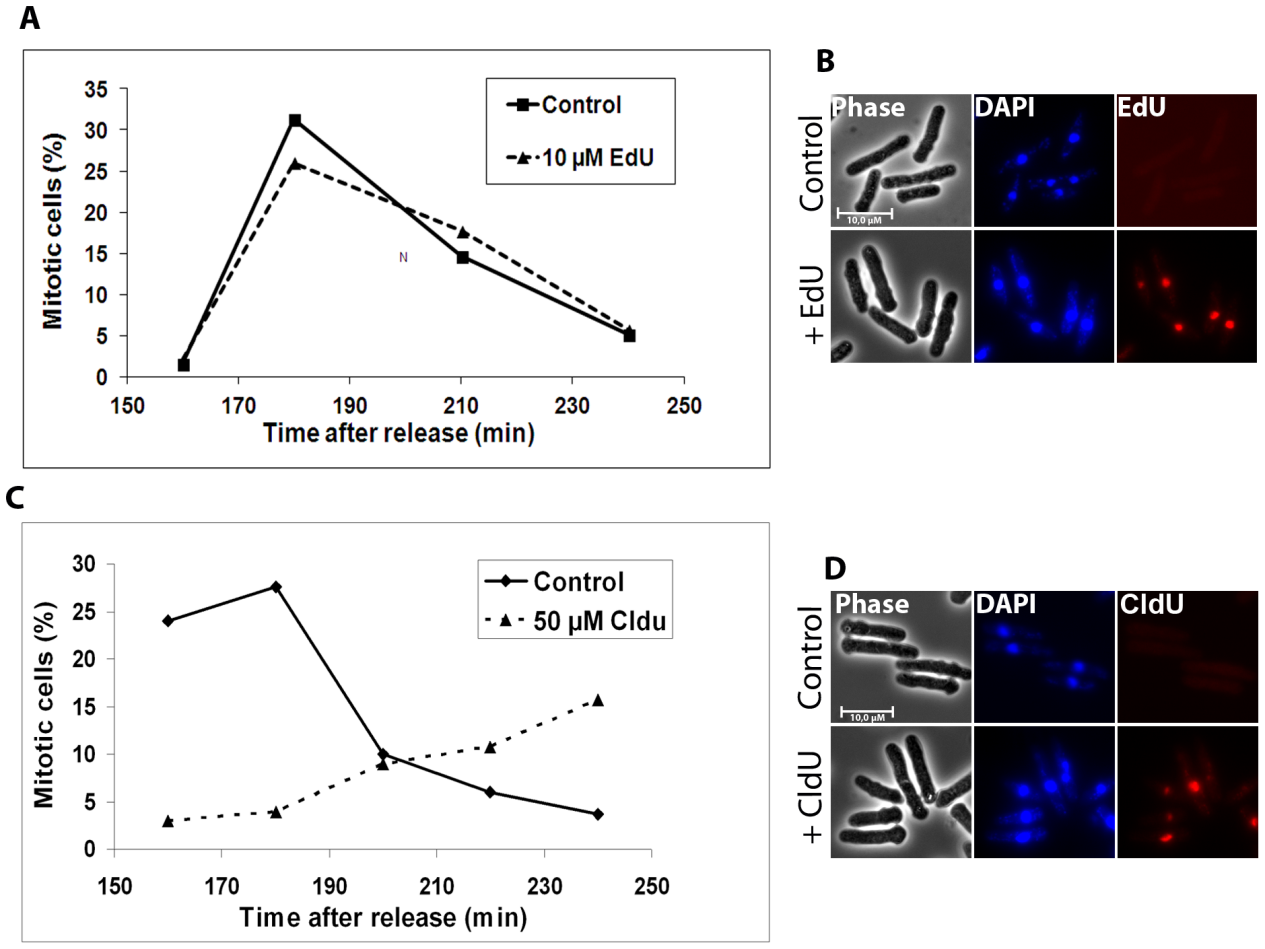
Short-term effects of EdU and CldU on cell-cycle progression. Cells synchronized in G1 phase were released and pulse–labelled with either CldU or EdU. The cells were harvested at the indicated time points, fixed, stained with DAPI and visualized using a fluorescence microscope. **A)** Percentage of mitotic, EdU-labelled cells at different time points after release. 200 cells were counted at each timepoint. **B)** Images of unlabelled control cells (upper panel) and cells labelled with EdU (lower panel) 160 minutes after release are shown to confirm that the analogue did incorporate into the DNA. **C)** As in A, but with CldU-labelling. **D)** As in B, but with CldU-labelling.

The EdU-concentration recommended by the manufacturer (10 µM) is 5-fold lower than the optimal concentration for CldU (above). We reasoned that EdU and/or the fact that it can be used at lower concentration might affect the cell cycle less severely than CldU. Cells synchronized in G1 were pulse-labelled with either 10 µM EdU or 50 µM CldU to compare the effects of the two analogues. Sixty minutes after release, the cells were washed to remove the analogues from the medium, incubation was continued, the samples were fixed at different time points and stained with DAPI. Cell-cycle progression was scored by counting mitotic cells in a microscope. EdU-labelled cells showed the same cell-cycle kinetics as unlabelled cells (Fig. 2A, B) indicating no checkpoint activation. On the other hand, for the cells that had incorporated CldU, the cell-cycle kinetics was affected when compared to untreated cells (Fig. 2C, D). Similar to our conclusion that EdU affects the first cycle to a lesser extent than CldU, it was recently showed that BrdU-labelled cells complete S phase after release from an HU block more slowly than EdU-labelled cells [16]. However, in these experiments they did observe an effect on S-phase progression also after EdU-labelling, in contrast to our results. The main difference in the two experiments is that they labelled the cells after an HU arrest, whereas untreated cells were labelled in the current work. HU depletes the nucleotide pools and thus most likely sensitizes the cells to a nucleoside analogue and indeed, they showed that in the reverse experiment BrdU labelling sensitizes the cells to HU [16].

We conclude that 10 µM EdU, at least when present for only 1 hour, does not significantly affect the following mitosis. However, 50 µM CldU does affect cell-cycle progression. It is important to note that CldU was used at a concentration 5 times higher than that of EdU. However, lower CldU-concentrations (10 µM) are not sufficient for detection of DNA synthesis by fluorescence microscopy. This does not mean that EdU is less toxic than halogenated analogues if used at the same concentrations. However, if we compare toxicity at the analogues’ respective detectable concentrations, EdU is the least toxic analogue since it can be detected at lower concentrations. Therefore, we suggest that EdU-labelling using 10 µM for the duration of S phase is the method of choice when studying events within one cell cycle.

**Figure 3:**
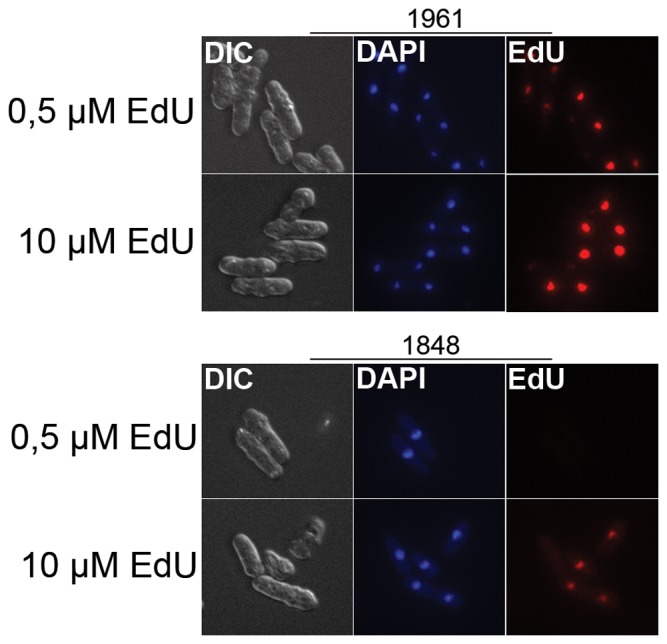
Comparison of labelling efficiencies of different strans. Exponentially growing 1961 (Rhind lab, upper panel) and 1848 (Forsburg lab, lower panel) were labelled with EdU at the indicated concentrations for 60 minutes and processed in parallel.

Using the Rhind construct, 0.5 µM BrdU and CldU [20] as well as 1 µM EdU [15] have been successfully used to label the DNA for DNA-combing experiments and even for whole-cell imaging [7]. To exclude differences in sample preparation and detection method, we have directly compared the labelling efficiency of the two strains. We confirmed that replicating DNA can be detected using 0.5 µM EdU in the strain from the Rhind lab and the intensity of the labelling was comparable to that using 10 µM EdU in the strain from the Forsburg lab (Fig. 3). We reason that the two constructs have clonal variations and have different labelling efficiencies.

### Long-term Effects of EdU- and CldU-labelling

**Figure 4:**
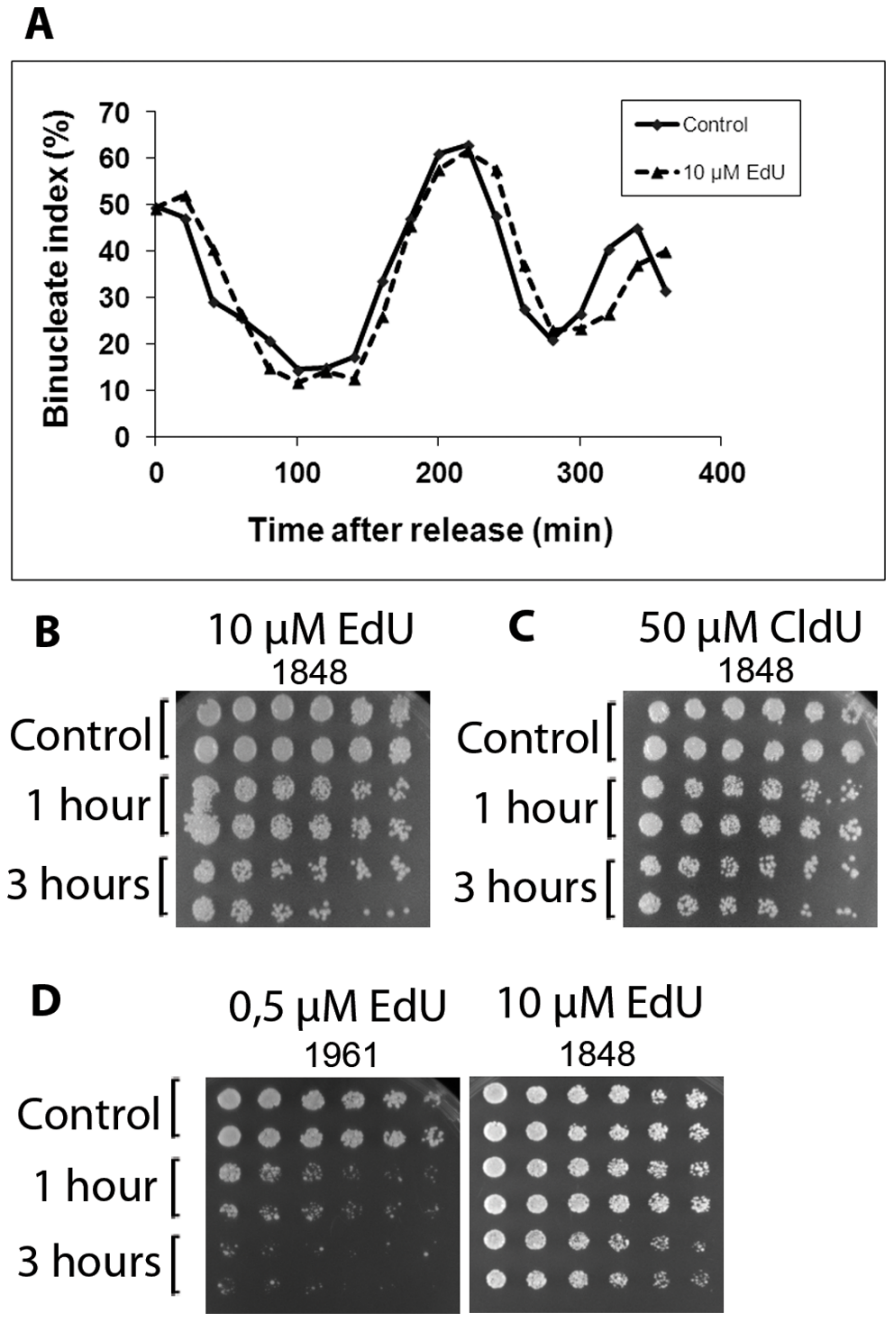
Cell survival after short- and long-term incubation with EdU and CldU. **A)** Effect of EdU through two cell cycles. Cells synchronized in G1 phase were released and labelled for 1 hour with EdU. The cells were fixed, stained with DAPI and analyzed by flow cytometry (10 000 cells at each timepoint). The graph shows the percentage of binucleate cells (M-G1), at the indicated time points, after release into the cell cycle. **B)** Cells arrested in G1 phase were released in the presence of 10 µM EdU. The analogue was removed after 1 or 3 hours, as shown. The cells labelled for 1 hour were incubated for a total of 3 hours before being plated. The cells were then plated onto YES plates in a 2× serial dilution and incubated at 25°C for 3 days. Cells incubated without EdU served as controls. **C)** As in B, but the cells were treated with 50 µM CldU or no CldU as control. **D)** Comparison of the survival of the two strains 1961 and 1848 (derived from 1947 (Rhind lab) and 1402 (Forsburg lab) after treatment with EdU at the indicated concentrations.

EdU was earlier reported to have an effect on cell viability [15]. Although we observed no significant differences between control and EdU-labelled cells during the first cell cycle (above), problems may arise in the next cell cycle(s). We investigated whether the subsequent cell cycle might be adversely affected by EdU-incorporation. The experiment was repeated as described above, and cell-cycle progression was scored by counting binucleate index [18] both in the first and the second mitosis after release and labelling. The kinetics of the first mitosis of EdU-labelled and unlabelled cells were similar (Fig. 4A). However, the second mitosis was slightly delayed in the EdU-labelled cells as compared to unlabelled control cells (Fig. 4A). Consistently, Sabatinos et al observed a more severe effect on the second S phase than on the first one after release from a HU block in the presence of EdU [16]. We speculate that the cells may have problems replicating the EdU-labelled DNA and thus the DNA-damage checkpoint might be activated in the second cell cycle. Previous work has shown that thymidine analogues cause phosphorylation of Chk1, which indicates that the DNA-damage checkpoint is activated [16].

The length of time that the analogue is present in the medium might have an effect on cell survival. To investigate this, cells grown in EMM were synchronized in G1 and upon release 10 µM EdU or 50 µM CldU was administered for 1 hours or 3 hours. The analogue was removed and the cells were plated to assay survival. The cells labelled for 1 hour were incubated for a total of 3 hours before being plated. To control that EdU was taken up by most cells during the 1 h incubation, a sample was taken 20 minutes after washing out the analogue, and the number of EdU positive cells was determined. 95% (n = 213) of the cells were EdU positive demonstrating that most cells have taken up the analogue during the 1 h incubation. The duration of the labelling clearly affected cell survival (Fig. 4B, C). For both analogues, a one-hour labelling resulted in lower survival than observed for unlabelled cells and a three-hour labelling resulted in an even lower survival. To ensure that the further reduction after three-hour labelling was not influenced by EdU being incorporated in the second S phase we measured the timing of the second S phase. To this end, we added EdU at 2 hours after release from a *cdc10* block and harvested samples at 3, 4 and 5 hours after release. EdU incorporation was only observable after 4 hours (data not shown), which demonstrates that the cells have not yet reached S phase three hours after release. We conclude that the detrimental effects of the analogues can not be solely explained by incorporation into the DNA. Consistently, BrdU has been shown to affect the cell-cycle progression by a mechanism not related to its incorporation into the chromosomal DNA [16]. With increasing BrdU-concentrations, the effects on cell-cycle progression became more severe, even when the amount of BrdU incorporated into the DNA was saturated [16].

Since different concentrations of EdU is required to detect DNA synthesis in the two strains deriving from the Forsburg and Rhind labs, we compared the effects of EdU on cell survival in the two strains. Cells were synchronized in G1, then they were released into the cell cycle and exposed to the concentrations at which the labelling could be detected (10 µM and 0,5 µM,) for 1 and 3 hours. Both strains survived better if the labelling was limited to 1 h as opposed to three hours, confirming the above results. Furthermore, the survival of the strain from the Rhind lab at 0.5 µM was lower than that of the strain from the Forsburg lab at 10 µM (Fig. 4D) even though the intensity of labelling is comparable (Fig. 3). Thus, more efficient labelling, meaning detectable labelling at lower analogue concentration in the medium, is not necessarily better when considering the overall effect on the cells. This result appears surprising in light of the above results showing that it is important to use the lowest possible analogue concentrations. However, the toxic effect of the analogues is most likely determined by how much analogue is imported into the cells and how much is incorporated into the DNA. These parameters, in turn, are determined by the activity and expression level of the transporter and the thymidine kinase.

Taken together, thymidine analogues have an effect on cell-cycle progression when they are (i) incorporated into the chromosomal DNA and (ii) present in the cells also outside of S phase. These results clearly demonstrate the importance of using the lowest analogue concentration that allows detection in the particular construct being used and of minimizing the time the analogue is present in the medium.

#### EdU can be used for early detection of entry into S phase

**Figure 5:**
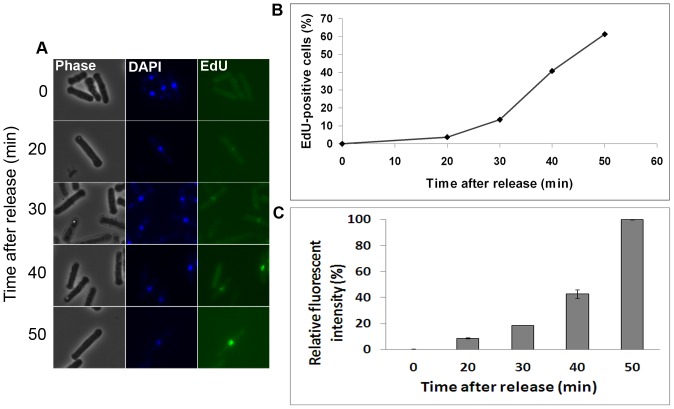
Early detection of S phase using EdU labelling. Cells synchronized in G1 phase were released in the presence of EdU, harvested at the time points indicated, fixed, and imaged by fluorescence microscopy. **A)** Fluorescence microscopy images of EdU-labelled cells at the indicated time points. **B)** Illustrates the percentage of EdU-labelled cells, at different time points after release. 200 cells were counted at each timepoint. **C)** Graph illustrating the relative fluorescent intensity between the samples indicated. The fluorescence intensity was measured using ImageJ 1.46 r. The background was set to the intensity measured at 0 min. The intensity measured in the 50 min sample was set to 100%.

We addressed whether S phase can be detected at an earlier time point using EdU-labelling than can be done by DNA measurements using flow cytometry. Cells synchronized in YES were released in the presence of 10 µM EdU and samples were harvested every ten minutes. Already at 20 minutes after release a weak EdU-specific signal could be observed from a few cells by fluorescence microscopy (Fig. 5A). The fraction of cells showing EdU-incorporation increased with time (Fig. 5B), probably reflecting the degree of asynchrony in S-phase entry and progression. The strength of the fluorescence signal from individual cells increased with time (Fig. 5C), as could be expected from cells traversing S phase. These results demonstrate that DNA replication can be detected already at 20 minutes after release from a G1 block, which is at least 20 minutes earlier than can be achieved by using flow cytometry (Fig. 1).

**Figure 6:**
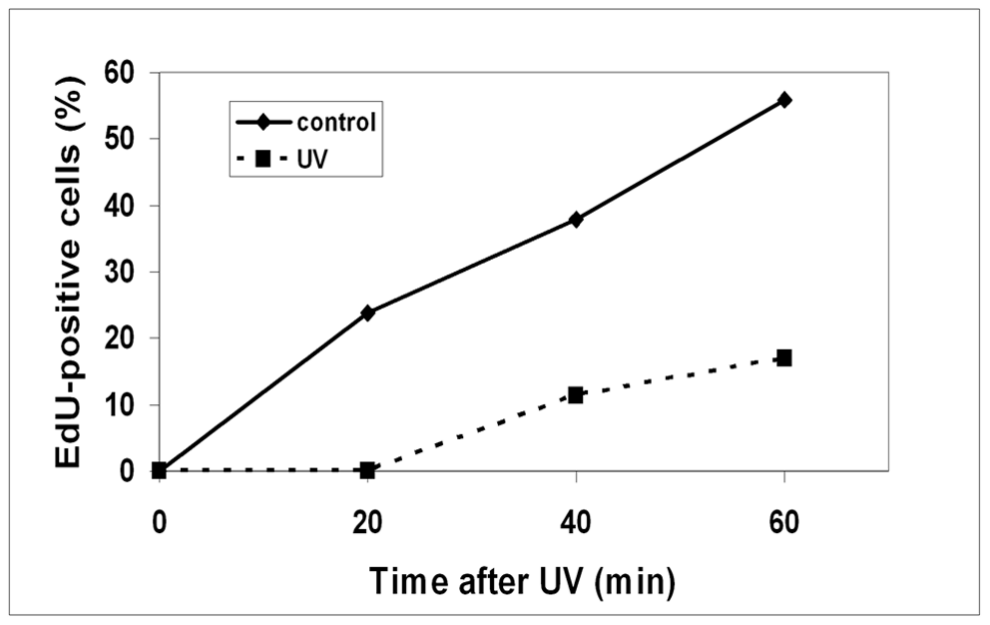
Monitoring cell-cycle progression in an asynchronous culture. Asynchronously growing cells were irradiated with 1100/m^2^ to a survival of >90%. Immediately after irradiation EdU was added and the samples were fixed, processed and imaged by fluorescence microscopy. The graph illustrates the percentage of EdU-labelled cells at the indicated time points. 100 cells were counted at each timepoint.

We also investigated whether EdU can be used to detect S phase in asynchronous cells. We have previously shown that when cells synchronized in G1 are exposed to UV-irradiation, entry into S phase is delayed [21]. Here we UV-irradiated exponentially growing cells and investigated whether we can detect the S-phase delay. EdU was added to a final concentration of 10 µM immediately after irradiation with 1100 J/m^2^. Samples were harvested at the indicated time points after UV-irradiation (Fig. 6). We observed a gradual increase in EdU-labelled cells in the control cells, but in the UV-irradiated cells EdU-incorporation could be detected only at later time points, indicating a cell-cycle delay. Since any synchronization method disturbs the cell cycle, EdU labelling of asynchronous cultures might be a useful method to investigate cell-cycle progression.

**Figure 7:**
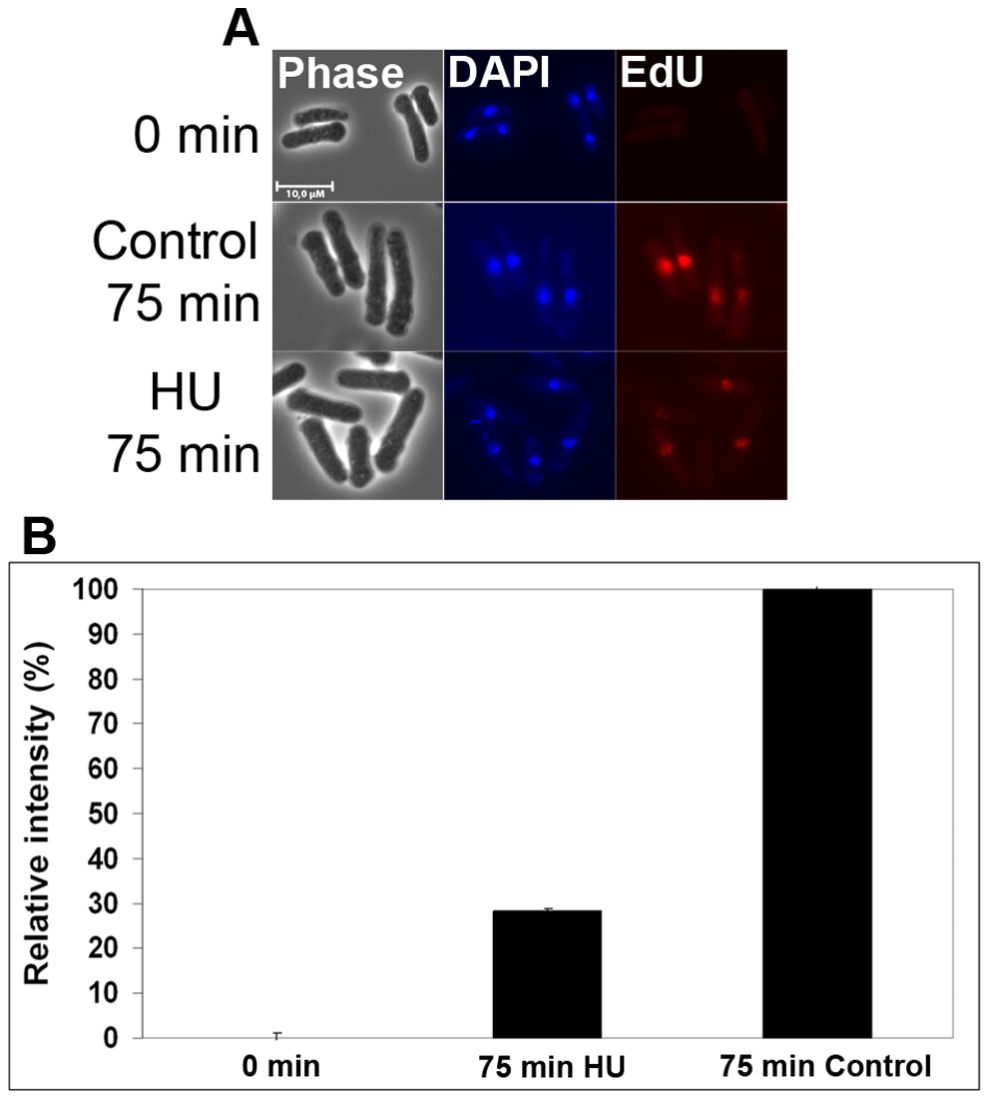
Detection of replication in HU-arrested cells. Cells synchronized in G1 phase were released in the presence of EdU or EdU plus HU. The cells were fixed after 75**A)** Fluorescence microscopy images of control cells at 0 minutes (top), EdU-labelled control cells at 75 minutes (middle) and EdU-labelled HU-treated cells at 75 minutes (bottom). **B)** Graph illustrating the relative fluorescent intensity in the samples indicated. The fluorescence intensity was measured using ImageJ 1.46 r in 50 cells at each timepoint. The background was set to the intensity measured at 0 min. The intensity measured in the 50 min sample was set to 100%.

Furthermore, we investigated whether newly-replicated DNA can be detected in HU-arrested cells. HU inhibits deoxyribonucleotide (dNTP) synthesis and the dNTP pools become exhausted shortly after early replication origin firing [22], [23], allowing only a limited extent of elongation. Cells grown in YES were synchronized in G1 and released in the presence of either 10 µM EdU or 15 mM HU plus 10 µM EdU. Cells were harvested upon release and after 75 minutes. Consistent with previous results [15], [24], incorporated EdU can be detected in HU-arrested cells (Fig. 7A), even though the signal is not as strong as in control cells, which progress further into S phase (Fig. 7B).

Taken together, these results demonstrate that labelling the DNA using EdU provides a sensitive method that can be used to detect low levels of DNA synthesis.

#### DNA repair synthesis after UV-irradiation

UV-irradiation causes DNA damage, mainly in the form of 6-4 photoproducts and cyclobutane pyrimidine dimers (CPDs). These lesions are excised by the nucleotide excision repair (NER) or UV-excision repair (UVER) pathways in fission yeast [25]. For each lesion, single-stranded stretches of about 30 nucleotides are synthesized [26]. In G1 phase, the excision-repair pathways NER and UVER are the only available repair pathways for UV-induced damage. This is in contrast to in G2 where recombinational repair can also be induced. We set out to investigate whether EdU incorporation can be used to detect excision-repair synthesis in G1 after UV-irradiation in fission yeast.

**Figure 8:**
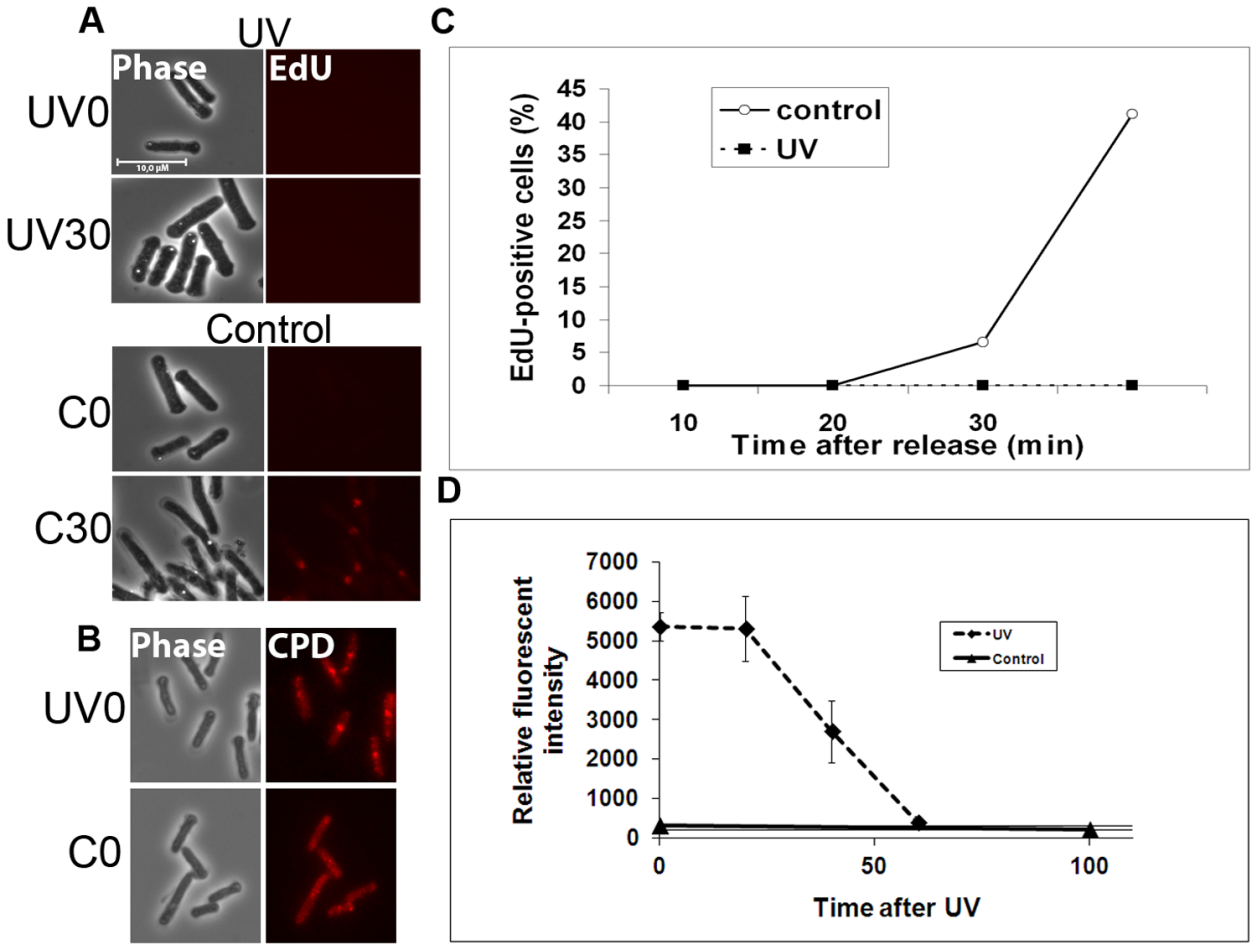
Detection of repair synthesis with EdU. Cells arrested in G1 phase were released in the presence of EdU and immediately UV-irradiated with 1100 J/m^2^. Control cells were not irradiated. Samples were harvested at the time points indicated, fixed, processed, and imaged by fluorescence microscopy. **A)** Microscopy pictures of EdU -labelled UV-irradiated and control cells 0 and 30 minutes after release. **B)** Detection of CPD-s by indirect immunflourescence in control and UV-irradiated cells immediately after irradiation. **C)** The graph shows the percentage of EdU-positive cells in UV-irradiated and control cells at the different time points after release. **D)** The graph shows the relative fluorescence intensity of CPDs as measured by indirect immunfluorescence in UV-irradiated and control cells at the different time points after release.

Cells synchronized in G1 were released into EMM containing 10 µM EdU and immediately UV-irradiated to 10–20% survival. As a control, cells were released into EMM with 10 µM EdU, but without UV-irradiation. These control cells showed the S-phase kinetics and EdU signals 20 and 30 minutes after release (Fig. 8) as described above. For the UV-irradiated cells, however, no EdU incorporation could be detected for any of the time points earlier than 40 minutes (Fig. 8). We did not expect to detect any replicative DNA synthesis to occur in the UV-irradiated cells at these times because they are arrested in G1 by UV-irradiation, thus delaying the onset of S phase [21]. To confirm that DNA repair does take place during the first 40 minutes, the presence of CPD-s, the major form of UV-induced damage, was detected by fluorescence microscopy. Over half of the lesions is repaired by 40 minutes (Fig. 8D), indicating efficient excision repair. Our results clearly demonstrate that EdU-labelling does not allow, under these conditions, the detection of DNA repair synthesis. Furthermore, this lack of detection confirms our previous data demonstrating a G1/S checkpoint in *S. pombe* induced by UV light. We have previously shown that this dose of UV-irradiation induces 0.2–0.3 CPD per kb of DNA [27]. Considering that the fission yeast genome is about 13,8 Mb and that a minimum of 30 nucleotides are synthesized for each CPD, we estimate that at least 10^5^ nucleotides can be incorporated after UV-irradiation. This is apparently not enough to be detected by labelling with 10 µM EdU. Since we could detect EdU-incorporation in HU-arrested cells, but not after repair of damage caused by UV-irradiation, there was most likely more DNA synthesis occurring in HU-treated cells than in the UV-irradiated cells.

### Sequential Labelling with Two Different Analogues

A double-labelling technique can be used to discriminate between the DNA synthesis occurring at different times during the same S phase or occurring in consecutive S phases [20], [28], [29]. This technique has been used successfully for several organisms and cell lines [7], [30], [31]. Labelling of two consecutive S-phases using IdU and CldU has been done in fission yeast for DNA-combing experiments [20]. However, we find that the analogue concentrations used in those experiments are too low for immunofluorecent detection in whole cells. To label the DNA in two generations is particularly challenging if the label arrests or perturbs the cell-cycle progression. BrdU, CldU and IdU are all detected by indirect immunofluorescence, so that detection of these labels can be combined as long as there are differentially labelled antibodies available. Since EdU has a less severe effect on the cell cycle than the halogenated analogues (above), combining EdU labelling with any of the other analogues is preferential to combining two halogenated analogues. More recently, combination of EdU and BrdU has been successfully used for DNA-combing experiments [24].

Here we show that the DNA can be labelled in two successive S phases using two different analogues, EdU and BrdU, and their presence detected in fixed cells. BrdU is detected by indirect immunofluorescence and EdU is detected by direct fluorophore conjugation, so that detection of these labels can be combined.

**Figure 9:**
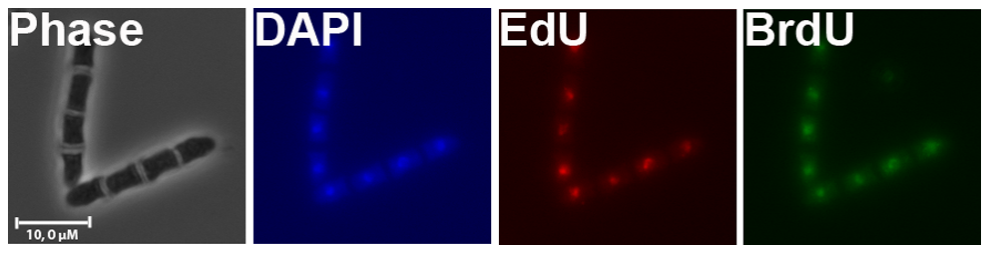
Sequential labelling using EdU and BrdU. Microscopy pictures of cells double-labelled with EdU and BrdU. Cells synchronized in G1 phase were released and pulse-labelled in two consecutive S-phases, first with EdU, then with BrdU, as described in the text. Samples were harvested after the next mitosis, fixed and processed as described and imaged using fluorescence microscopy. Negative controls for cross-reactivity are provided on Fig. S3.

Cells growing in YES medium were arrested in G1 phase, released in the presence of EdU and 1 hour later the analogue was removed to minimize the time of exposure (see above). One doubling time after release, BrdU was added to label cells in the second S phase and the analogue was removed after 1 hour. Samples were harvested after the next mitosis had taken place, when septa appeared. The cells used in this experiment contained a mutation (*sep1*) that prevents the separation of daughter cells, so that after two cell cycles, four granddaughter cells are attached and can be easily recognized [32]. Figure 9 shows fluorescence images of cells where the DNA was sequentially labelled with EdU and BrdU.

Using this method it is possible to differentially detect both EdU and BrdU incorporation, without cross-reactivity (Fig. S3), in two successive S phases. This system can be used to track the fates of single DNA strands over several generations, making it possible to distinguish “old” from “new” strands (to be published elsewhere).

## Conclusions

Here we have optimized the conditions for labelling the DNA of fission yeast cells with thymidine analogues, for the use in cell-cycle studies. Specifically, we have investigated the short- and long-term effects of such labelling. Furthermore, we show that labelling with analogues can be used for early detection of S-phase entry. By using low concentrations and short labelling pulses to reduce the adverse effects of the analogues we have demonstrated the feasibility of DNA labelling with two distinct thymidine analogues in two sequential cell cycles. These advances will contribute to more detailed and accurate cell-cycle analyses in particular when using fission yeast as a model organism.

## Supporting Information

Figure S1 **Flowcytometry analyses of cell-cycle progression.** DNA measurements of cells arrested in G1 and released into the cell cycle. Stained cells were analyzed based on area (DNA-A) and pulse width (DNA-W) of the Sytox Green fluorescence signal. Two-parametric DNA cytograms (left) with indicated positions for the G1- and S/G2- cells and one-parametric DNA histograms (right) are shown.
(TIF)

Figure S2 **Titration of CldU.** Fluorescence micrographs of cells grown in the presence of the CldU-concentrations indicated. The cells were synchronized in G1 phase, released and labelled for 1 hour before fixation, and analysis by fluorescence microscopy. There are two separate experiments represented in this figure. First we tested 790, 380, 190 and 95 µM CldU and found all the concentrations sufficient to detect the DNA. The next experiment was done with 50, 10 and 0 µM EdU.
(TIF)

Figure S3 **Microscopy pictures of cells labelled with EdU or BrdU.** Cells were labelled with either analogue and detection for both analogues was performed to check cross-reactivity.
(TIF)
