# Supplementary figures and images for: Cell-Cycle Analyses Using Thymidine Analogues in Fission Yeast
Source: PLoS One. 2014 Feb 13;9(2):e88629. doi: 10.1371/journal.pone.0088629 (PMC3923809; doi:10.1371/journal.pone.0088629)

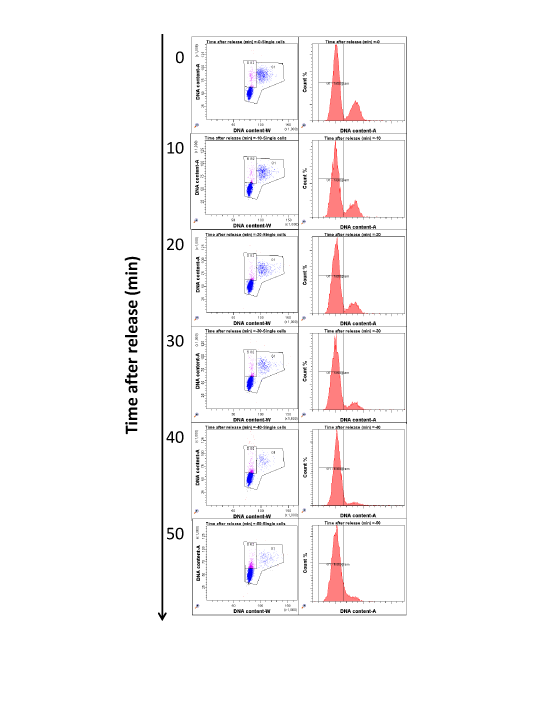

Supplement: Figure S1 — Flowcytometry analyses of cell-cycle progression. DNA measurements of cells arrested in G1 and released into the cell cycle. Stained cells were analyzed based on area (DNA-A) and pulse width (DNA-W) of the Sytox Green fluorescence signal. Two-parametric DNA cytograms (left) with indicated positions for the G1- and S/G2- cells and one-parametric DNA histograms (right) are shown. (TIF) [file pone.0088629.s001.tif]

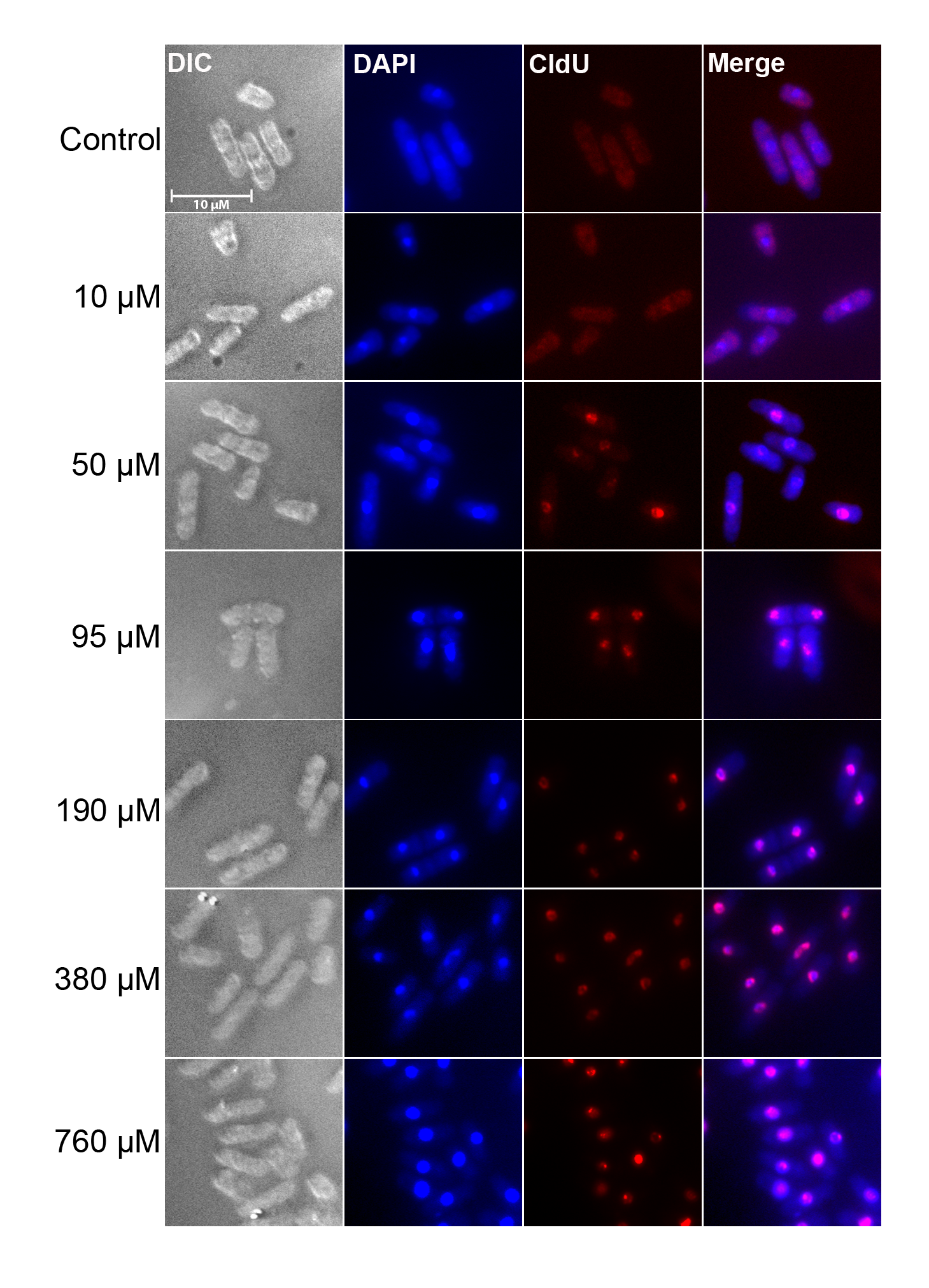

Supplement: Figure S2 — Titration of CldU. Fluorescence micrographs of cells grown in the presence of the CldU-concentrations indicated. The cells were synchronized in G1 phase, released and labelled for 1 hour before fixation, and analysis by fluorescence microscopy. There are two separate experiments represented in this figure. First we tested 790, 380, 190 and 95 µM CldU and found all the concentrations sufficient to detect the DNA. The next experiment was done with 50, 10 and 0 µM EdU. (TIF) [file pone.0088629.s002.tif]

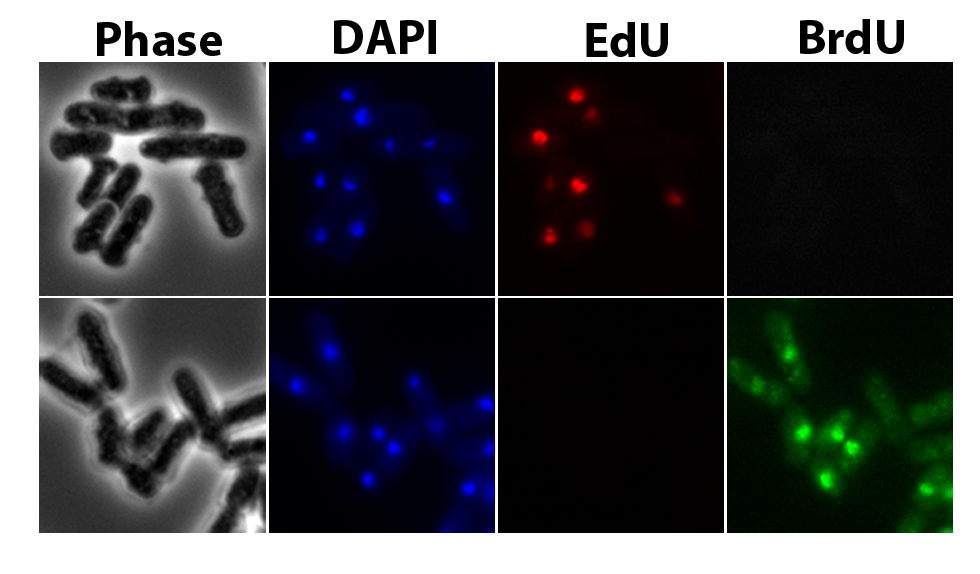

Supplement: Figure S3 — Microscopy pictures of cells labelled with EdU or BrdU. Cells were labelled with either analogue and detection for both analogues was performed to check cross-reactivity. (TIF) [file pone.0088629.s003.tif]
